# Spraying of Ultrathin Isoporous Block Copolymer Membranes—A Story about Challenges and Limitations

**DOI:** 10.3390/membranes10120404

**Published:** 2020-12-07

**Authors:** Thomas Bucher, Juliana Isabel Clodt, Clarissa Abetz, Barbara Bajer, Volkan Filiz

**Affiliations:** Helmholtz-Zentrum Geesthacht, Institute of Polymer Research, Max-Planck-Str. 1, 21502 Geesthacht, Germany; thomas.bucher@pall.com (T.B.); clarissa.abetz@hzg.de (C.A.); barbara.bajer@hzg.de (B.B.); volkan.filiz@hzg.de (V.F.)

**Keywords:** isoporous membrane, block copolymer, pH responsiveness, spray coating, upscaling, ultrafiltration

## Abstract

Isoporous membranes can be prepared by a combination of self-assembly of amphiphilic block copolymers and the non-solvent induced phase separation process. As the general doctor-blade technique suffers from high consumption of expensive block copolymer, other methods to reduce its concentration in the casting solution are sought after. Decreasing the block copolymer concentration during membrane casting and applying the block copolymer solution on a support membrane to obtain ultrathin isoporous membrane layers with e.g., spraying techniques, can be an answer. In this work we focused on the question if upscaling of thin block copolymer membranes produced by spraying techniques is feasible. To upscale the spray coating process, three different approaches were pursued, namely air-brush, 1-fluid nozzles and 2-fluid nozzles as generally used in the coating industry. The different spraying systems were implemented successfully in a membrane casting machine. Thinking about future development of isoporous block copolymer membranes in application it was significant that a continuous preparation process can be realised combining spraying of thin layers and immersion of the thin block copolymer layers in water to ensure phase-separation. The system was tested using a solution of polystyrene-*block*-poly(4-vinylpyridine) diblock copolymer. A detailed examination of the spray pattern and its homogeneity was carried out. The limitations of this method are discussed.

## 1. Introduction

Amphiphilic block copolymers (BCP) are in a specific focus of membrane scientists since they can be used in a fast membrane casting process combining their self-assembly ability and the non-solvent induced phase-separation process (SNIPS) [1,2,3,4,5,6,7,8]. The isoporous hexagonal [9] or square pore lattice [3] on top of the membrane is directly formed within the process, an advantage compared to other methods for the formation of isoporous membranes. The thin selective top layer is merging in a sponge-like substructure underneath. The most understood diblock copolymer system in this respect is polystyrene-*block*-poly(4-vinylpyridine) (PS-*b*-P4VP) diblock copolymer, firstly reported in 2007 [1,9,10,11,12]. BCP membranes can be post-modified in order to implement additional characteristics or to tailor the pores [13,14,15,16,17,18], which will bear potential for various application if scalability is possible. One important limitation of the scalability and usability of these types of membranes is the high consumption of the expensive block copolymer in common doctor-blade casting, even if the polymer concentration could be reduced by the inclusion of additives or blending [19,20,21]. Moreover, it was clearly pointed out that the substructures of PS-*b*-P4VP membranes play an important role with regard to their separation performance [22]. The substructure resistance of BCP membranes limits the transport rate and leads to higher adsorption. One possibility to overcome this problem could be to decrease the membrane thickness to a minimum, leading to research in this direction. In order to reduce the amount of BCP necessary for the membrane formation and to reduce substructure resistance, Hahn et al. combined spray respectively dip coating with the block copolymer self-assembly on support membranes followed by non-solvent induced phase inversion process in 2015 [23]. The formed thin film isoporous block copolymer membranes were stable at least up to 2 bar transmembrane pressure and showed pH responsive behaviour when the pH sensitive BCP, PS-*b*-P4VP, was used. The method was successful for different BCP and on different support but up to now limited to a few cm^2^. Later on, a roller coating process was investigated to apply our approach of saving BCP which could reduce the BCP consumption by over 95% [24]. Furthermore, block copolymer ultrafiltration membranes were described by Ma et al. using a combination of spray coating of PS-*b*-P2VP and selective swelling [25]. Even cationic and anionic diblock copolymer nanoparticles were sequential spray coated to fabricate nano-structure membranes [26]. In Layer-by-Layer (LbL) assembly of multilayer thin films, spray coating is common and superior [27] to other techniques as a quick process [28] that can be transferred to larger surfaces using common systems of the coating industry [29].

**Figure 1:**
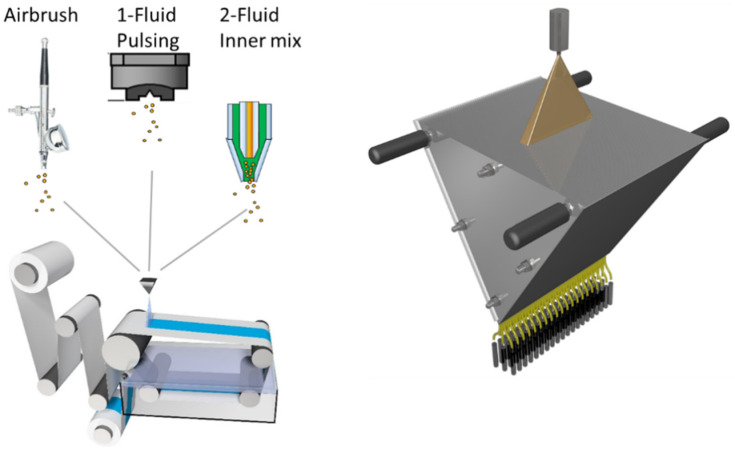
Schematic presentation of the membrane production process with three different spraying techniques combined with a membrane casting machine including a non-solvent bath (**left**) and spray collector (**right**).

In this work we focused on the question if upscaling of thin BCP membranes produced by spraying techniques is feasible. Using spraying techniques, the consumption of BCP will be decreased compared to the common doctor-blade technique. This is a fundamental aspect thinking about possible industrial application of isoporous BCP membranes in the future. One important objective was the selection of a system that could be used to achieve homogeneous isoporous membrane surfaces over large membrane areas in a continuous process. For the first time, BCP membranes were made using spraying devices implemented to a membrane casting machine bearing a non-solvent precipitation to allow the phase-separation process. The membrane casting machine is generally used for doctor-blade technique and was adjusted for this study. To upscale the spray coating process, three different approaches were pursued and a schematic overview is shown in Figure 1. The simple air-brush technique, commercial 1- and 2-fluid nozzles as used in the coating industry were tested in this process. A detailed examination of the spray pattern examined with a spray collector (Figure 1, right) and a discussion about limitations of the spraying technique itself were carried out, since the latter might be less familiar to membrane scientists.

## 2. Materials and Methods

### 2.1. Adjusted Membrane Casting Machine

**Figure 2:**
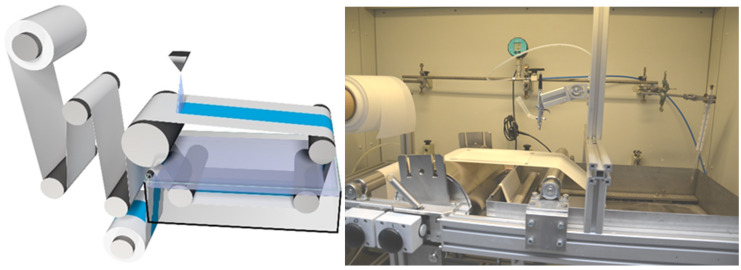
Schematic view of the in-house casting machine for lab-scaled membrane casting (**left**) and image (**right**).

In order to upscale the preparation of thin isoporous block copolymer membranes by spray coating, our in-house designed casting machine used for general membrane casting via SNIPS was adapted to a spray coating device. An airbrush pistol or a spraying device was attached perpendicular to the support as shown in Figure 2. The spraying device and its controller were purchased from Spraying Systems GmbH (Hamburg, Germany), connectors and pipes from Swagelok Comp. (Solon, OH, USA), pressure valves from Festo SE & Co. KG (Esslingen, Germany) and pressure measurement devices LEO3 from Keller AG (Winterthur, Switzerland). The spraying device consists of three parts: the automatic spraying nozzle, which can be pulsed by opening and closing the spraying nozzle with an electromagnetic plunger up to 10,000 times per minute, a controller device which adjusts the speed of the plunger, and the exchangeable spraying nozzle-tips (head of spraying nozzle), which are responsible for the different spray patterns-, angle- and flow rates.

### 2.2. Spraying Nozzles

We used a 1-fluid-spraying nozzle with six different tips listed in Table 1. 1-Fluid spraying nozzles were chosen in this upscaling attempt in order to save polymer. This means only pressure is applied to the fluid and the geometry of the nozzle causes the liquid stream to collapse into a fine spray without the need of a gas, like in 2-fluid spraying nozzles (e.g., spraying can or air brush). The big advantage is the theoretical saving of polymer, because in comparison to 2-fluid nozzles, there is much less overspray. Mass loss also called overspray means mass that is not attaching to the support, e.g., due to the gas stream that blows away the liquid. The 1-fluid nozzles chosen in this work are listed in Table 1. The 1-fluid nozzles can be used to pulse the spray by quickly opening and closing the spraying nozzle. The mass flow reduction by pulsing is further called pulsing rate and is tuneable from 0%–100% where 0 means a fully closed and 100% a fully open spraying nozzle without pulsing. In between, the controller unit calculates the optimum pulsing frequency to reduce the flow rate according to set value, while highest possible pulsing frequencies are preferred for this purpose to keep the spraying pattern as constant as possible. The spraying pattern should ideally not be influenced, but the mass flow is reduced. The nozzles differ in their orifices and in their “response characteristic” which means the impulse within the nozzle channel. After a plunger quickly opens and closes the channel, the induced impulse is slightly smoothening with increasing channel lengths. The mass loss of the produced spray also differs with each nozzle depending on selected spraying parameters. The 2-fluid nozzles used in this work contain a liquid and a service gas which generate a fine spray with fine droplets, also called atomising the fluid stream. The service gas gives them their impulse into a specific direction influenced by the nozzle orifice. The mixing of the gas stream with the liquid stream can be within the spraying nozzle, called inner mixing or on the outer part, when a liquid stream comes out of the nozzle and is converted into an aerosol at the outside by applying a strong gas stream next to it. In the case of outer mixing, the applied pressure in the tank of the liquid stream is much smaller, than the pressure of the gas stream. Inner mixing needs slightly higher liquid pressures to overcome the overpressure of the mixing chamber to avoid backflushing into the liquid line. Generally, the inner mixing can produce a finer spray, but the flow rate is sensitive to small pressure changes within the mixing chamber and therefore more difficult to adjust.

In order to save polymer, 1-fluid spraying nozzles were chosen first for our purpose instead of 2-fluid nozzles, due to smaller mass losses of their spray.

### 2.3. UV Measurements

For UV measurements an 8 W UV-lamp (Camag; Berlin, Germany) was used at 254 nm wavelength with a camera EOS D60 (Canon; Krefeld, Germany) for imaging in manual mode with the settings f-number F5.6, ISO1000, focal lengths 18 mm and shutter speed 1 s, carried out in a Camag UV-cabinet (Camag; Berlin, Germany). We increased the contrast between the PAN-support membrane and the PS-*b*-P4VP top layer by using a UV-lamp (254 nm). While the PAN backscatters parts of the UV-light, which then appears purple, PS-*b*-P4VP partially absorbs this wavelength and appears black. If the PS-*b*-P4VP layer thickness is low the UV-light will penetrate this layer, backscatter at the PAN-support membrane, and not absorb completely on its way back. In consequence, deviations of PS-*b*-P4VP layer thickness can be seen as purple light with changing intensity. To gain this effect, the shutter speed of the camera was increased to 1 s, which was enough for our purpose.

### 2.4. Spray Collector (Patternator)

**Figure 3:**
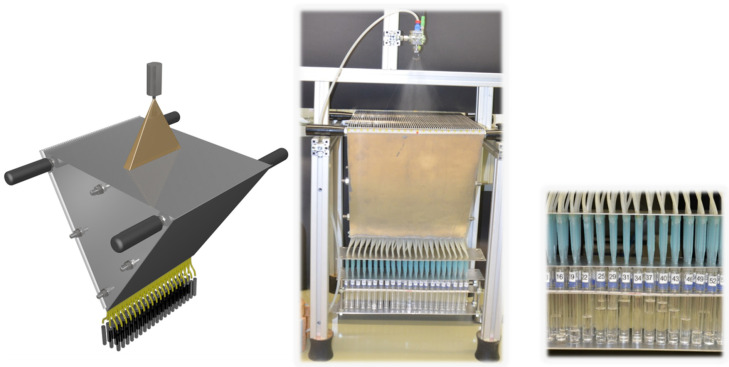
Schematic view (**left**) and image (**center**, **right**) of an in–house spray collector. Spray is collected in 66 chambers, each of 0.47 cm width; 31 cm in total. The spraying nozzle was fixed on an aluminium profile rack (Rose + Krieger GmbH; Minden, Germany) at a distance of 17.5 cm. Testing tubes were arranged in three rows.

Quantities of the flow rate were analysed by collecting the sprayed liquid with a spray collector, also called patternator, depicted in Figure 3. Herein the sprayed liquid is collected at different positions below the nozzle in 66 chambers with connected tubes, each chamber of 0.47 cm width and 40 cm long. For this experiment the distance of the nozzle to the chamber was set to 17.5 cm. In order to compare the flow rate of the different nozzles, pure water was used as a liquid.

Each tube was weighed before and after spraying for a defined time. The flow rate at each position was calculated as

(1) $$q_{m}=\frac{\Delta m}{\Delta t}$$

where *q_m_* is the flow rate and Δ*m* is the mass collected inside a tube over a period of time Δ*t* (Equation (1)).

### 2.5. Preparation of Block Copolymer Membranes

The block copolymer PS-*b*-P4VP was synthesised via sequential living anionic polymerization following a synthesis route according to Rangou et al. [9]. All membranes were spray-coated with solutions of 1 wt% PS_83_-*b*-P4VP_17_^88k^ (M_w_ 88 kg/mol, 17 wt% P4VP, 83 wt% PS) dissolved in 1,4-dioxane (Sigma Aldrich, St.Louis, MO, USA), stirred overnight until the solution appeared homogenous. We kept the polymer concentration constant, to focus on the investigation of the spraying parameters and the setup. This polymer concentration and polymer composition showed good results in previous studies [23] using hand-held airbrush technique for the casting step followed by a manual transport into the precipitation bath.

The block copolymer solution was spray-coated with an in-house casting machine on two different support membranes, namely polyacrylonitrile (PAN, HZG) [30] or polyvinylidene difluoride (PVDF, HZG) [31] membranes, using either an airbrush gun type Sogolee AB-430 (Conrad electronic SE; Hirschau, Germany) with a nozzle diameter of 0.3 mm or different nozzles purchased from Spraying Systems GmbH (Hamburg, Germany) as described above.

### 2.6. Scanning Electron Microscopy (SEM)

A Merlin and an LEO 1550 VP (both Zeiss, Oberkochen, Germany) were used for scanning electron microscopic investigations of the membranes at accelerating voltages of 3 kV or 5 kV, respectively. Secondary electrons were used to image the morphology. The samples were previously sputter coated with approximately 2 nm platinum. Cross-sections were prepared by first infiltrating the porous membranes with iso-propanol, then freezing the samples in liquid nitrogen and finally breaking them. Average pore size values were determined using the software IMS, V15Q4, from Imagic Bildverarbeitung AG (Glattbrugg, Switzerland) on the basis of the SEM results.

### 2.7. Permeance Measurements

Permeance measurements and pH-dependent permeance measurements were performed in dead-end mode using an in-house automatic testing device at transmembrane pressures of 2.0 bar to 2.1 bar at room temperature. These studies were conducted with demineralized water with an electrical conductivity of ≈0.055 μS·cm^−1^. The effective membrane area was 2.35 cm^2^. HCl was used to set the desired pH stepwise between pH 5 and pH 3. For each pH value the membranes were rinsed for 20 min at 2.1 bar transmembrane pressure to reach equilibrium followed by a permeance measurement over 10 min. The permeance, also called normalized water flux, (*P*) was calculated by normalizing the flux by the transmembrane pressure

(2) $$P\;=\frac{\Delta V}{A\;\Delta t\;\Delta p}$$

where Δ*V* is the volume of water collected between two mass measurements, *A* is the membrane surface area, Δ*t* is the time between two mass measurements, and Δ*p* is the transmembrane pressure (Equation (2)).

## 3. Results and Discussion

### 3.1. Upscaling of Sprayed Block Copolymer Membranes with an Airbrush Pistol

**Figure 4:**
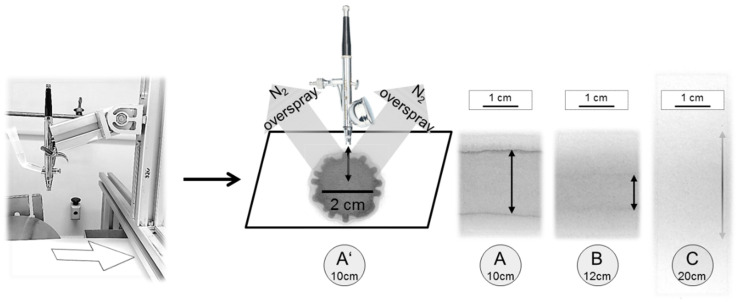
Airbrush pistol fixed on an aluminium profile rack (Rose+Krieger GmbH) with self-build tank. Solvent was 1,4-dioxane with small amounts of black ink added to the solution. (**A’**) illustrates sample sizes with conditions similar to previous works (~3 cm²) [23]; distance d from nozzle to support was 10 cm, pressure p was 1.5 bar. In (**A**–**C**) we started moving the support with 8 mm/s slightly raising the distance. (**A**) with d = 10 cm shows a dense homogeneous film of ~2 cm, (**B**) with d = 12 cm the width of the dense homogenous part falls to ~1 cm and (**C**) with d = 20 cm it was not possible to get a dense film because of too quick solvent evaporation.

Our first successful attempts to prepare thin block copolymer membranes were carried out with an airbrush pistol and other methods [23,25]. In order to upscale this process we combined the airbrush technique with our in-house casting machine [9]. Firstly, the dispersion of a liquid on the support and different adjustments for the setup were analysed by simply spraying black ink dissolved in 1,4-dioxane, i.e., the solvent for PS-*b*-P4V, on a support. When the airbrush pistol was attached perpendicularly to the support with a distance of 10 cm, we found a maximum diameter of 2 cm in stationary mode as shown in Figure 4A’. In previous studies it was found that an evaporation time of 5–10 s was optimum for the formation of the membrane. When the support was moved with a speed assuring such an evaporation time, we obtained a line of about 1.5 cm diameter and homogenous coverage (Figure 4A). We expected to cover more area, when raising the distance to 12 cm, which was the case, but the area where the droplets really overlap and form a dense homogenous film gets smaller, in this case about 1 cm width (Figure 4B). Raising the distance up to 20 cm (Figure 4C) leads to a gradient-like film with highest concentration directly under the nozzle and decreasing toward the edges, where the film is not dense according to visual inspection. This effect is caused by the evaporation of solvent before reaching the support. Slowing down the support speed did not lead to better films. Without droplet overlap, it is impossible to get a dense, homogenous film. In consequence self-assembly does not lead to a membrane with an isoporous top layer [32].

**Figure 5:**
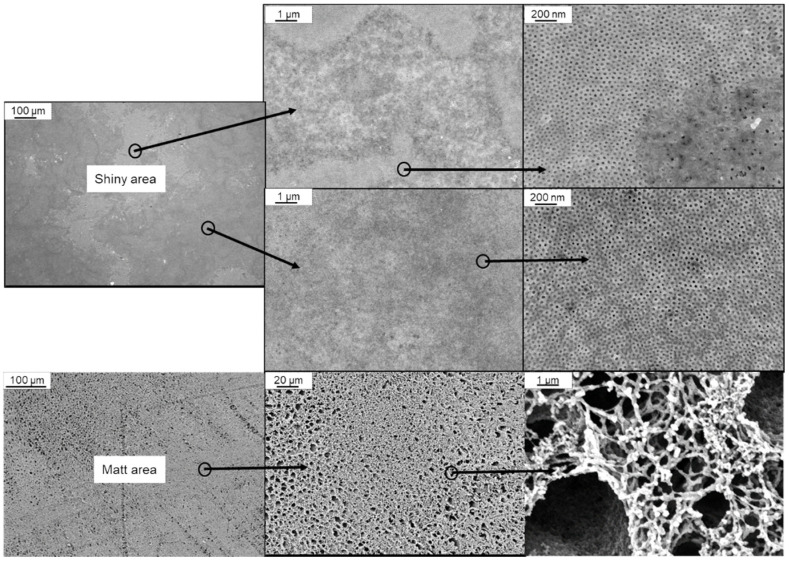
SEM images of BCP membranes sprayed on a supporting PAN-membrane with an airbrush system using 1 wt% of PS_83_-*b*-P4VP_17_^88^ solution in 1,4-dioxane. Magnification of different parts of the main shiny area with partial typical pores of BCP membranes, and the matt area without hexagonally arranged pores are shown (bottom).

When a BCP solution is sprayed with an airbrush pistol combined with our casting machine working continuously below 5 cm·s^−1^, strong wavemarks can occur on the membrane, that break the membrane surface after drying since the BCP membrane is very thin. On the other hand, increasing the casting speed to avoid wavemarks and keeping the evaporation time between 5–10 s led to insufficient amount of BCP on the surface of the support membrane and inhomogeneity as shown in Figure 5. Both shiny and matt areas occur as membrane surfaces, with the former making up the majority of the area. These parts show mostly hexagonally arranged pores that can be open or closed, but also disordered parts without any pores. The matt part does not show any ordered pore structure (Figure 5, bottom).

**Figure 6:**
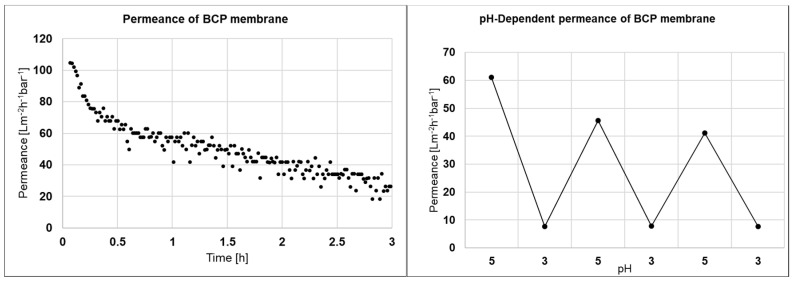
Permeance of a BCP membrane made from PS_83_-*b*-P4VP_17_^88^ solution in dioxane sprayed with airbrush on a PVDF support using a membrane casting machine (**left**) and pH-dependent permeance of the same membrane (**right**).

BCP membranes made from PS-*b*-P4VP are able to close their pores between pH 3 and pH 5 due to swelling of the poly(4-vinylpyridine) block. Therefore the permeance and the pH-dependent permeance of the main shiny areas were measured and the results are depicted in Figure 6. The permeance decreases from above 100 to 20 Lm^−2^ h^−1^ bar^−1^ over 3 h. Although we did not investigate the decline further, we assume that fouling, swelling, compaction or a sum of these effects to be possible reasons. Furthermore, residual loosely polymer agglomerates from the phase inversion process may block some inner pores during permeance measurements. The switchability between pH 3 and pH 5 is still detectable with a permeance above 40 Lm^−2^ h^−1^ bar^−1^ at pH 5 decreasing below 10 Lm^−2^ h^−1^ bar^−1^ at pH 3. The switchability confirms at least for the shiny area a membrane without cracks on the surface

A disadvantage of the membrane are the defects without pores or closed pores on the surface referring to low permeances. Using airbrush and hand casting, in short we observed four times higher permeances in previous studies without the casting machine [23]. An explanation for this finding is the limitation of the casting speed and the amount of spray coated on the support while the support is moving, which could be solved by using other spraying techniques with higher mass flow than airbrush. While in the common doctor-blade technique almost the complete polymer solution is fixed on the support material, spraying techniques suffer from overspray, which is matter that is not attached to the surface and blown away. Also, 2-fluid nozzles like air-brush pistols show strong overspray. The width of homogenous coverage may be strongly limited.

Furthermore the permeances are a bit lower than permeances of PS-*b*-P4VP membranes with similar pore sizes prepared by blade casting [22].

We assume that upscaling spray-coated BCP membranes in a continuous process, using airbrush in combination with a membrane casting machine cannot yield BCP membranes with sufficient morphology and homogeneity in dimensions useful for industrial application. Simultaneously, the new technique should exhibit advantages compared to the blading techniques.

### 3.2. Upscaling of Sprayed Block Copolymer Membranes with 1-Fluid Nozzles

#### 3.2.1. General Remarks

**Figure 7:**
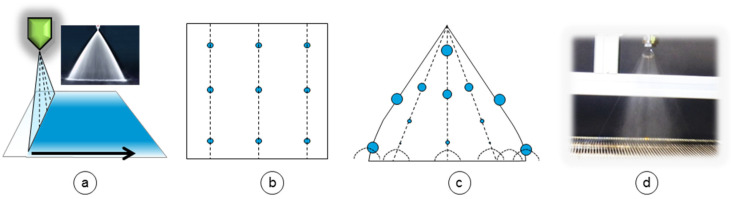
Illustration of the spraying nozzle setup (**a**) (lateral view), an ideal spray (**b**) (medial view), a real spray (**c**) (medial view), and a photograph of one of the used spraying nozzles (**d**) (medial view).

1-fluid spraying nozzles are used in the coating industry, are only pressure driven, and have much less overspray than airbrush in principal. The mass flow of 1-fluid nozzles is much higher compared to airbrush. They can be purchased as flat spray nozzles promising a spray pattern of homogenous coverage over a large width of membrane support (Figure 7a).

The spray pattern is still influenced by many aspects, like the nozzle geometry, applied pressure, liquid viscosity, surface tension, temperature of the liquid and the spraying angle. Additionally, the droplet velocity quickly decreases after complete spray formation due to friction forces depending on its droplet sizes and shapes leading to fog- or rain-like sprays. Figure 7b depicts a homogenous ideal spray in frontal plane (medial view). Dashed lines mark droplet flight path. In real spray, the droplet size differs over the width (Figure 7c,d) whereas at the edges the spray is stronger with bigger droplets due to the geometry of the nozzle orifice. The droplet speeds and consequently evaporation of solvent is also not the same over flight time at different positions (Figure 7c).

#### 3.2.2. Spray Pattern of 1-Fluid Nozzles

**Figure 8:**
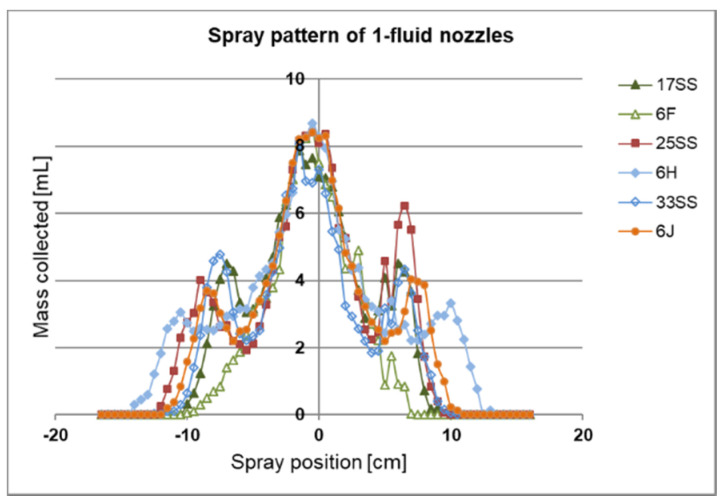
1-Fluid-nozzles spraying pattern. The spraying pattern along the y-axis was measured with a spray collector. The x-axis shows the mass distribution of the spray collected in at least 66 testing tubes at different positions. The pressure was set to *p(L)* = 2.5 bar with a distance of *d*(*NS*) = 17.5 cm. The humidity was 44%, and room temperature was 21.6 °C.

For our 1-fluid nozzle approach we purchased different spraying nozzles as listed in Table 1. In order to analyse the spray distribution beyond the nozzles we built a spray collector (Figure 3). All 1-fluid nozzles were tested at a pressure of the liquid *p(L)* = 2.5 bar and a distance to the spray collector of *d*(*NS*) = 17.5 cm (distance from nozzle to support). Using this distance the best analysis was possible. The results are shown in Figure 8.

The liquid mass remained at a similar level at distances of approximately ±2 cm from the nozzle, placed on 0 cm position of the x-axis. Beyond ±2 cm, the liquid mass decreased to approximately 25% for all nozzles, then increased again to 50% with the exception of the 6F until the spray reached its edge at around ±10 cm apart from the nozzle. The spraying pattern was nearly symmetrical. The deviation beyond ±2 cm apart from the nozzle is too high for a larger-scale flat sheet membrane manufacture.

#### 3.2.3. Adjusting the Mass Flow

For our application, the mass flow of 1-fluid nozzles had to be adjusted, because it was too high. In general the mass flow could be decreased by decreasing the nozzle size, the pressure or by increasing the distance to the support and the speed of the casting machine. Even if we chose the smallest 1-fluid flat sheet nozzles available (6F), the mass flow was still too high. By decreasing the pressure the spray pattern is affected (droplet size, angle, film homogeneity). There is an increase of the volumetric flow rate with increasing pressure between 1 and 3 bar, but at least approximately 1.5 bar is required for the spray to become fine enough to cover the surface accurately without obvious bigger droplets. On the other hand, the mass flow can easily exceed a threshold value beyond which the wet film is turned into a swimming state. A pressure of 2.5 bar was a good compromise between those two states. By increasing the distance *d(NS)* between the nozzle and the support, the spraying pattern is also affected when the distance gets too large. Here, 17.5 cm turned out to be close to an empirical optimum in case of water, which means the nozzle was not too close to the support. The mass per unit area can be decreased by increasing the speed of the support in our casting machine, but this is also technically limited since the BCP solution needs a defined evaporation time for the self-assembly before the non-solvent induced phase separation occurs.

**Figure 9:**
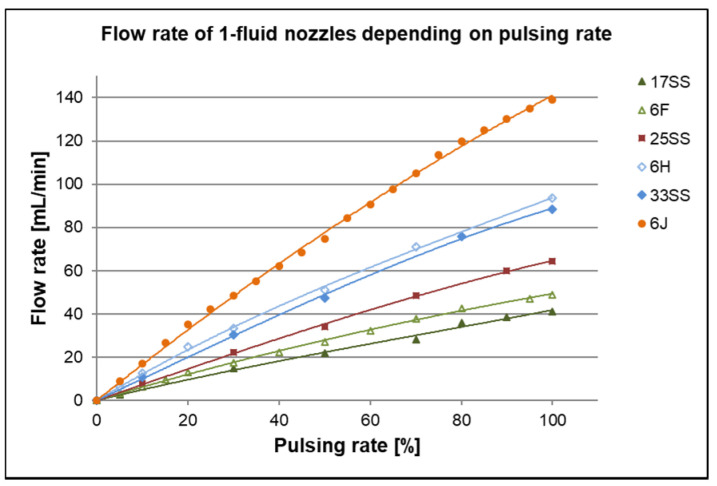
Flow rate of each spraying nozzle at different pulsing rates, measured at 2.5 bar with 1,4-dioxane. 100% means mass flow at fully opened spraying nozzle without pulsing.

Another way to decrease the mass flow of spraying is the pulsed mode of the 1-fluid nozzles. Herein the orifice is opened and closed in a cycle up to 15,000 pulses per minute. This strongly reduces the flow rate, without changing other parameters. All spraying nozzle flow rates were tested at different pulsing rates with 1,4-dioxane as liquid and at 2.5 bar. The results are depicted in Figure 9.

As expected, the flow rates increase with increasing size of the equivalent orifice of the nozzles and with the pulsing rate. Generally, the flow rate increases almost linearly with increasing pulsing rate. There is only a little reduction of the slope of flow rate vs. pulsing rate for pulsing rates between 80%–100%. The equivalent orifices of the nozzles 6F and 17SS, respectively 6H and 33SS, have the same size by manufacturer information (Table 1) which means, the flow rate should be the same. As shown in Figure 9, we found a deviation of ~16% from 17SS to 6F and ~5% from 33SS to 6H for the mass flow without pulsing (equates to 100% pulsing rate), which indicates the limits of precision of very small spraying nozzles.

The spraying pattern was affected in all cases of pulsing. We observed bigger droplets on the samples, than in non-pulsed mode. These droplets adversely distribute on the membrane surface so that defects are possible. The big droplets might occur at the moment when the nozzle is opening again after being closed in pulsing mode. However, pulsing was necessary for our purpose in order to achieve the optimum mass flow for the preparation of BCP membranes.

#### 3.2.4. Spraying BCP with 1-Fluid Nozzles

For this test, 1 wt% solutions of PS_83_-*b*-P4VP_17_^88^ dissolved in 1,4-dioxane were sprayed using 1-fluid nozzles on a PAN membrane used as support and immersed in water.

**Figure 10:**
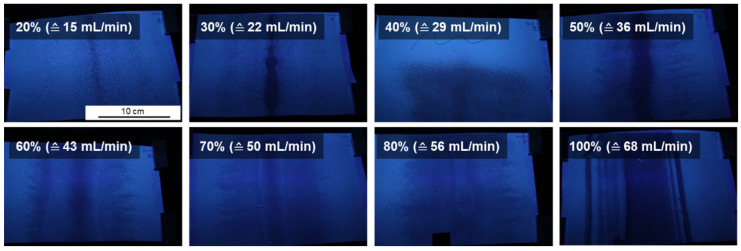
Spray cast membranes of PS_83_-*b*-P4VP_17_^88k^ at different pulsing rates *R* under UV-light after casting and drying (25SS).

Most of the membranes appear inhomogeneous under daylight. In order to make the inhomogeneity of the membranes visible on a larger scale we made photographs under UV-light. Figure 10 shows spray cast membranes exemplarily at different pulsing rates, while the pressure, spraying angle and support speed were kept at constant speed. The film thickness increased with the pulsing rate.

**Figure 11:**
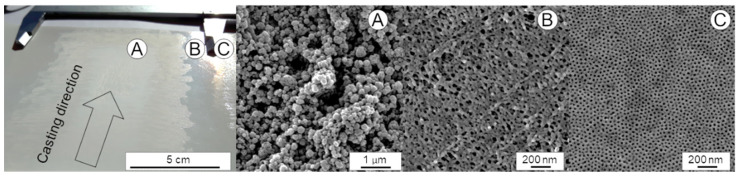
Spray cast sample of PS_83_-*b*-P4VP_17_^88^ solution in 1,4-dioxane with a 1-fluid nozzle (6H), 2.5 bar, distance from support to the nozzle 17 cm, casting speed 18 mm/s, pulsing rate 15%, evaporation time 18 s. The spraying pattern gradually changed from a thick, amorphous matt film in the middle part (**A**) into a shiny film next to it (**B**) and another shiny section but with different reflection (**C**).

For all experiments using different nozzles and pulsing rates we observed inhomogeneity of the sprayed films. These deviations are macroscopic and could be seen without magnification. They become visible using light reflection as different shiny or matt areas. SEM images of these different parts were prepared and the results are depicted in Figure 11. The biggest part A, a matt area, does not show any pores but round agglomerates of polymer. The more shiny area B already shows small disordered pore-like parts. In the smallest part C the formation of a regular structure of hexagonally packed pores was observed with a medium pore diameter of 14.2 nm. This result is comparable to the structures of the previous small-scale study [23].

Permeances of below 50 Lm^−2^ h^−1^ bar^−1^ were measured for different membrane sheets of the small area C with hexagonally arranged pores The values were even lower than those for common BCP membranes made of PS_83_-*b*-P4VP_17_^88^ that are generally in the region of 70–100 Lm^−2^ h^−1^ bar^−1^ [22]. On the other hand some of these sheets were just dense and permeances could not be measured.

#### 3.2.5. Challenges and Limitation of the 1-Fluid Nozzles Approach

For thin spray cast films, there is a high risk of inhomogeneous distribution of the sprayed material on the support. Even though all the 1-fluid spraying nozzles showed a fan jet patterning, strong deviations over larger distances could not be compensated by droplet overlapping. Moreover, the mass flow of the 1-fluid nozzles was too high for our purpose even at low pulsing rates. The higher mass flow at pulsing rate 100% improved the homogeneity within the centre area by forming a thick wet film, that more easily equilibrates deviations. But the necessary evaporation time of the solvent for this low concentration of BCP is too long with this continuous rolling-up approach of our casting machine with limits in dimension and speed. Decreasing the casting speed will cause strong wavemarks on the casted membranes as mentioned above. A casting machine with very long distances between the spray coat and the coagulation bath might overcome this problem. In order to reduce the mass flow similar to airbrush techniques that were successful at least for small membrane areas and hand casting, another approach (3.3) was carried out for the upscaling development.

### 3.3. Upscaling of Sprayed Block Copolymer Membranes with 2-Fluid Nozzles

#### 3.3.1. Homogeneity of the BCP Spray of 2-Fluid Nozzles

2-Fluid nozzles were tested as another attempt to upscale sprayed membranes similar to the airbrush approach applied successfully [23] to a 3 cm^2^ scale. A detailed description about these kinds of nozzles utilised for inner or outer mixing of liquid and gas is given in the experimental part.

**Figure 12:**
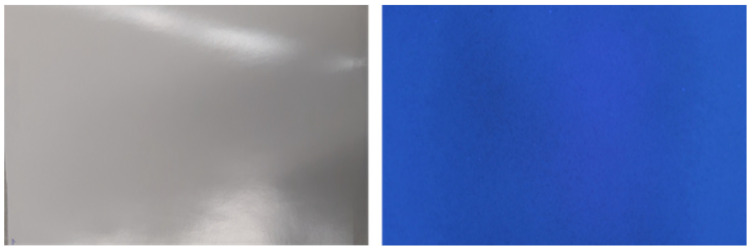
Spray cast membranes of PS_83_-*b*-P4VP_17_^88k^ with 2-fluid/inner mix nozzle, normal image (**left**) and image under UV-light (**right**) after casting and drying.

We first tested the 2-fluid/inner and 2-fluid/outer mixing nozzle regarding the mass flow. The 2-fluid/outer-mixing nozzles showed an easier control of the mass flow, because it is only dependent on the pressure of the liquid, *p(L)*. The atomisation takes place at the outer side and changing the gas pressure, *p(G)*, does not influence the mass flow of the liquid line. However, the gas stream had to be stronger in comparison to the 2-fluid/inner mix nozzle for a finer spray. The inner mixing nozzles showed a mass flow, that depends both on *p(L)* and *p(G)*. If *p(L)* becomes smaller than the pressure in the mixing chamber, the gas stream will penetrate the liquid line. The finer spray is produced with the inner-mixing nozzles and an adequate film homogeneity could be reached as shown in Figure 12.

The membranes appear homogeneous over a large area and even under UV-light no defects or inhomogeneities were found, which was a strong improvement compared to the membranes sprayed with 1-fluid nozzles (compare to Figure 11).

**Figure 13:**
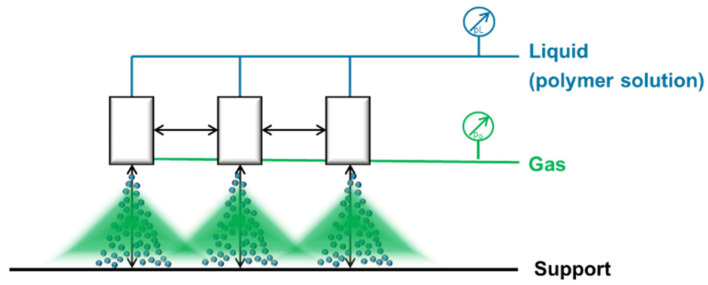
Setup for the overlapping of three 2-fluid nozzles. The support is moving perpendicular to the scheme.

Therefore we bought three nozzles of the same type to investigate the spraying pattern. Our aim was to overlap the spray distributed by these nozzles as shown in Figure 13 in order to upscale the width.

#### 3.3.2. Spray Pattern of 2-Fluid Nozzles

**Figure 14:**
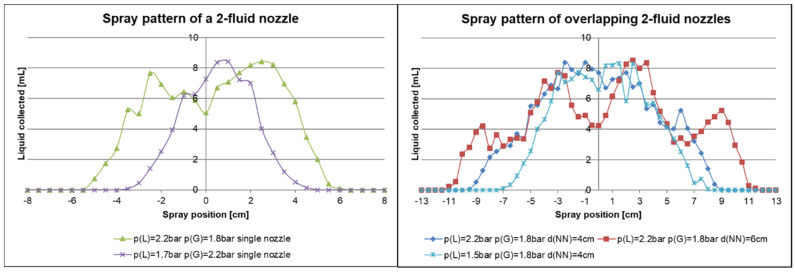
Spray pattern of a 2-fluid nozzle using different liquid pressure, *p(L)*, and gas pressure, *p(G)*, (**left**); Spray pattern of two 2-fluid nozzles overlapped using different pressures and distances of the nozzles, *d(NN)*, (**right**). The spraying pattern along the y-axis was measured with a spray collector. Water was used as the liquid and the nozzle to support distance was *d(NS)* = 15 cm.

In order to define a feasible setup for the overlapping, the spray pattern of a single nozzle was measured at different pressures and the results are depicted in Figure 14 (left). The spray pattern remained at a similar level approximately in the range of −1 cm to +2 cm with the nozzle at 0 cm, for *p(L)* = 1.7 bar and *p(G)* = 2.2 bar. Beyond the given range the amount of distributed spray decreased rapidly to 0. Changing the pressure to *p(L)* = 2.2 bar and *p(G)* = 1.8 bar refers to a broadening of the spray pattern as intended. It has to be mentioned that changing the liquid pressure as well as the gas pressure change the droplet size. This may result in big droplets that should be avoided to prevent inhomogeneity on the surface. Therefore, the spray pattern was only measured for reasonable adjustments of the pressure and not for those excluded by tests using water before. The spray pattern resulting from overlapping the sprays of two 2-fluid nozzles was measured with different distances of the nozzles *d(NN)*, and the results are depicted in Figure 14 (right). Here, 4 cm was the smallest distance that could be realized by the setup due to space limitation. Herein the spray pattern remained similar from approximately ± 4 cm and was broadened when the distance between the nozzles was increased to 6 cm. The *d(NN)* = 6 cm setup results in a more irregular pattern and we concluded that further separation of the nozzles will not improve the results. Changing the pressure of the liquid and the gas with *d(NN*) = 4 cm led to a smaller spray pattern. We decided to further investigate the spraying of a BCP solution in combination with our casting machine with *p(L)* = 2.2 bar, *p(G)* = 1.8 bar and *d(NN)* = 4 cm.

#### 3.3.3. BCP Membranes Sprayed with Three Overlapping 2-Fluid Nozzles

**Figure 15:**
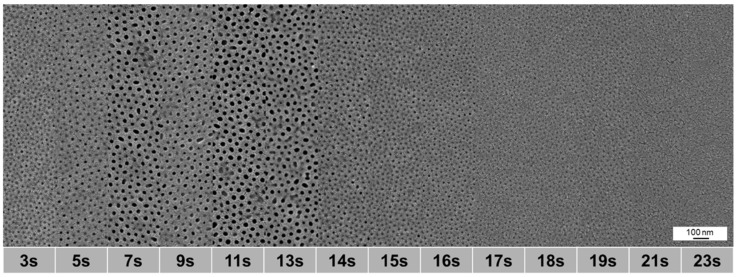
SEM images of spray cast samples of PS_83_-*b*-P4VP_17_^88^ solution in dioxane (1 wt%) with a 2-fluid nozzle and different evaporation times (from 3 to 23 s), *p(G)* = 1.8 bar gas, *p(L)* = 2.2 bar, *d(NS)* = 15 cm, *d(NN)* = 4 cm.

As the morphology of the membranes depends on the evaporation time of the cast block copolymer solutions before precipitation, we investigated different evaporation times from 3 to 23 s. Figure 15 shows that the evaporation time window for membranes with open pores is between 7 s and 14 s which fits to previous results [9,23]. The medium pore diameter ranges from 17.8 nm after 7 s to 19.8 nm after 11 s and decreasing again to 18.4 nm at 13 s of evaporation time in this region. After 11 s also the highest porosity of 10.9% was found. Compared to the preparation via airbrush including the casting machine or via 1-fluid nozzle, the structure appears homogeneous without defects.

**Figure 16:**
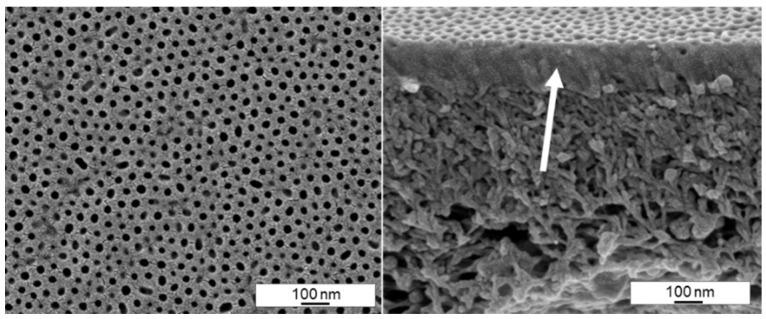
SEM image of the surface (**left**) and cross-section (**right**) of a spray cast sample of PS_83_-*b*-P4VP_17_^88^ solution in 1,4-dioxane (1 wt%) with a 2-fluid nozzle and an evaporation time of 11 s, *p(G)* = 1.8 bar gas, *p(L)* = 2.2 bar, *d(NS)* = 15 cm, *d(NN)* = 4 cm. The arrow points to the dense BCP part of the membrane, on top of the PAN support membrane.

We tried to measure the permeance of the membranes that show open pores according to the SEM images, but unfortunately all membranes were dense or almost dense with permeances below 5 Lm^−2^ h^−1^ bar^−1^. The reason for the impermeability is shown in Figure 16. Even though the pores appear initially open, a dense film of BCP was found in the cross section underneath. This dense film is around 150 nm thin and it appears that the block copolymer solution is partly blocking the PAN support membrane. We assume partly soakage of the solvent into the dry PAN support membrane after spraying, while the BCP-chains are retained on its top. In consequence the concentration of solvent quickly becomes very low at the PAN-interface hindering the formation of a spongy-like substructure within the BCP-layer. We were not able to solve the problem of the dense substructure yet. On the other hand, this was the thinnest film of BCP on a PAN support membrane that we could observe by spray coating. If a way is found ensuring the BCP substructure to stay open, the thickness of 150 nm could be a big advantage with respect to the membrane performance. In another study, the presence of nanofillers suppressed the formation of a dense layer between a block copolymer top layer and a porous support [33].

## 4. Conclusions

This work comprises three different approaches to upscale thin block copolymer membranes using spraying techniques. An airbrush pistol, 1-fluid nozzles and 2-fluid nozzles as generally used in the coating industry were installed in a membrane casting machine. The system was tested using a solution of polystyrene-*block*-poly(4-vinylpyridine) diblock copolymer and an examination of the spray pattern was carried out. The results can be summarized as follows:

In general, upscaling is possible using an airbrush pistol. Isoporous membranes could be formed with permeances of ~40 Lm^−2^ h^−1^ bar^−1^, but with a limitation of around 2 cm width when the airbrush pistol is connected to a membrane casting machine. The structure of the membrane is inhomogeneous and comprises defects without pores that do not influence the pH-sensitive behaviour of the membranes.

The spray pattern of 1-fluid nozzles was investigated and the best nozzle was selected including pulsing mode to influence the flowrate. The surfaces of membranes made with 1-fluid nozzles are inhomogeneous as observed by UV-light and have mostly undefined structures on the surfaces. Only on small parts the typical isoporous structure of BCP membranes was observed and low permeances from 0 to 50 L/m^−2^ h^−1^ bar^−1^ were measured.

With 2-fluid nozzles very thin films of around 150 nm thickness and a homogeneous surface were obtained including isoporosity. The window of evaporation time for the pore formation was studied and the best results were found for evaporation times in the range of 9–13 s. However, all membranes were almost dense and only very small or no permeances could be observed. The BCP solution appears to block the support membrane in this case.

In the future, an approach to prevent to some extent the penetration of BCP solutions into the support membrane is necessary. The use of fillers, other support material or an increased viscosity could be helpful. However, an increased viscosity will hinder the formation of small droplets in the spraying process. In this context, techniques like dip coating or profile roller coating seem to be more promising methods for upscaling the production of isoporous integral asymmetric block copolymer membranes.

## Figures and Tables

**Table 1:** Different spraying nozzles and equipment used in this work. Two nozzles have the same equivalent orifice *. A single 1-fluid nozzle and three equal 2-fluid nozzles in overlapping operation were used.

Component	Full Name	Type	Equivalent Orifice [mm^2^]	Abbreviation (Supplier)
Controlling unit	Autojet 2008 + PWM			
Automatic Spraying nozzle (1-fluid)	Pulsajet/BSPTAAB10000AUH-104210-EPR	1-fluid		
Nozzle-tip	TPU650017PWMD-SS	1-fluid	0.28 *	6F
Nozzle-tip	TPU650033PWMD-SS	1-fluid	0.38 *	6H
Nozzle-tip	TPU650050PWMD-SS	1-fluid	0.50	6J
Nozzle-tip	TPU650017-SS	1-fluid	0.28 *	17SS
Nozzle-tip	TPU650025-SS	1-fluid	0.33	25SS
Nozzle-tip	TPU650033-SS	1-fluid	0.38 *	33SS
Automatic Spraying nozzle (2-fluid) (3×)	AAB10000JJAU-VI	2-fluid		
Nozzle tip (liquid) (3×)	PFJ0850-SS	liquid cap		850
Nozzle cap (gas-ex) (3×)	PAJ105-50-SS	gas cap		Ext-mix
Nozzle cap (gas-in) (3×)	PAJ73328	gas cap		Int-mix

* These nozzles with equal equivalent orifice slightly differ in their channel lengths to the orifice which could have an effect on the spray pattern in pulsed mode. When the plunger opens and closes the impulse is slightly smoothened when the channel is longer. The focus of investigation was to generate the most homogenous spraying pattern.
